# Supplementary material for: Association between oral microbiome and seven types of cancers in East Asian population: a two-sample Mendelian randomization analysis
Source: Front Mol Biosci. 2023 Nov 21;10:1327893. doi: 10.3389/fmolb.2023.1327893 (PMC10702768; doi:10.3389/fmolb.2023.1327893)
Supplement: Supplementary file 1 [file DataSheet1.ZIP › Supplementary Materials/MR plots for tongue/tongue═╝/Gastric cancer/pheno.1644_to_gastric cancer_leave_one_out.pdf]

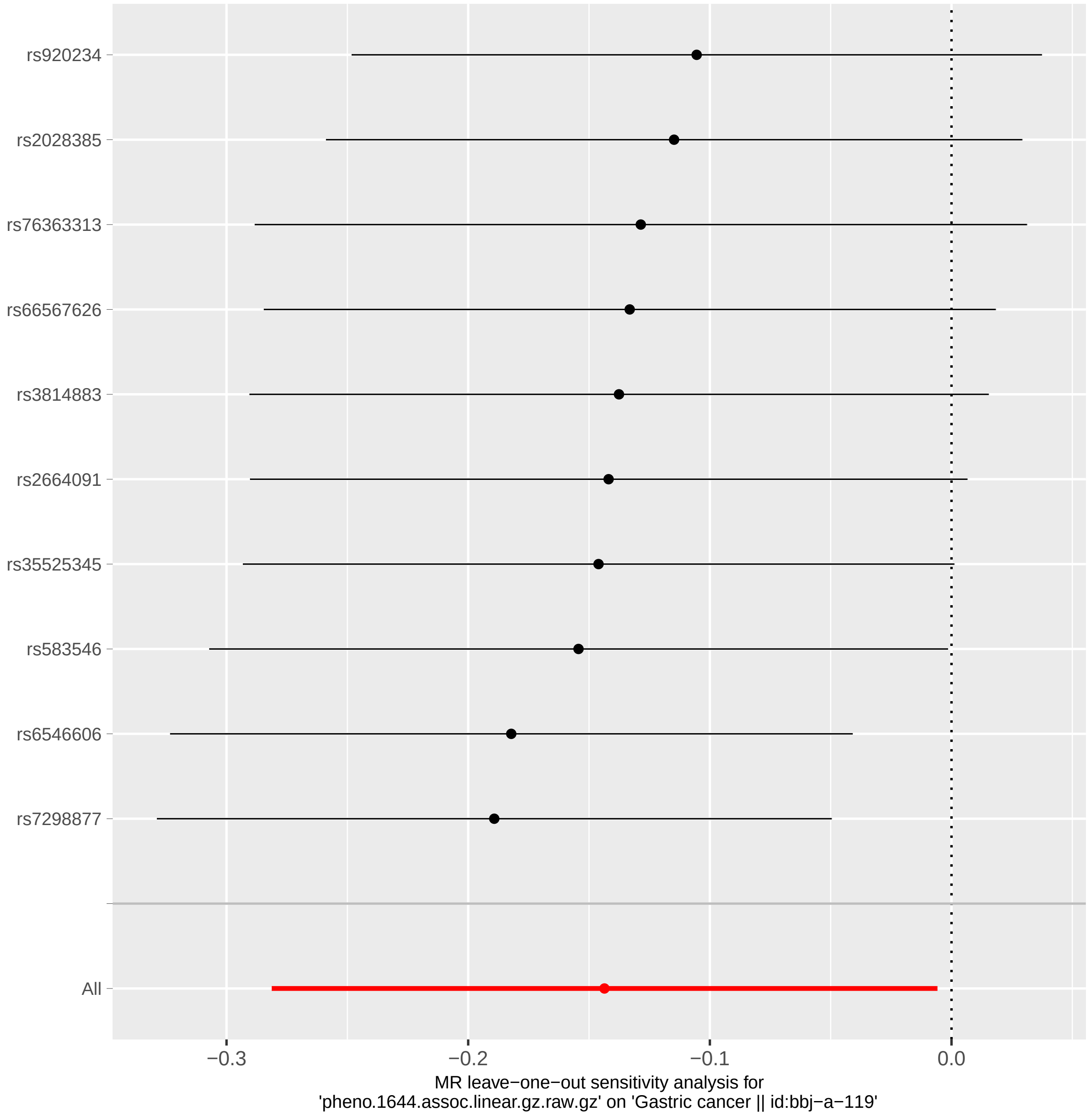

rs920234
rs2028385
rs76363313
rs66567626
rs3814883
rs2664091
rs35525345
rs583546
rs6546606
rs7298877
All
−0.3
−0.2
−0.1
0.0
MR leave−one−out sensitivity analysis for
'pheno.1644.assoc.linear.gz.raw.gz' on 'Gastric cancer || id:bbj−a−119'
